# Nonlinear association between pre-pregnancy body mass index and preterm birth in singleton pregnancies conceived with assisted reproductive technology

**DOI:** 10.3389/fnut.2025.1651462

**Published:** 2026-01-12

**Authors:** Tingting Zhuang, Jingli Sun, Yu Zhang

**Affiliations:** 1 Postgraduate Training Base of Jinzhou Medical University (General Hospital of Northern Theater Command), Shenyang, China; 2 Department of Obstetrics and Gynecology, General Hospital of Northern Theater Command, Shenyang, China

**Keywords:** pre-pregnancy body mass index, preterm birth, single birth, assisted reproductive technology, nonlinear

## Abstract

**Objective:**

This study aimed to analyze the correlation between pre-pregnancy body mass index (BMI) and the risk of preterm birth (PTB) in the assisted reproductive technology (ART) population and to determine the optimal BMI associated with the lowest risk of PTB.

**Design:**

Retrospective cohort study.

**Setting:**

The National Vital Statistics System birth dataset (January 1, 2019–December 31, 2023) from the National Center for Health Statistics in the United States.

**Subjects:**

For birth data from 2019 to 2023, women who conceived via ART, with a single live birth, age at delivery ≥18 years and gestational age at delivery of 24 to 41 weeks were included. Women with missing data were excluded. The study included a total of 197,237 women with an average age of 35 ± 5 years.

**Exposure:**

Pre-pregnancy BMI.

**Main outcome measures:**

The primary outcome is PTB (<37 weeks). Evaluate the relationship between pre-pregnancy BMI and PTB by adjusting the logistic regression model for confounding variables; using a 4-node restricted cubic spline (RCS) model to assess nonlinear associations; subgroup analysis was performed based on with or without previous termination or fetal loss; finally, sensitivity analysis was conducted to validate the robustness of the results.

**Results:**

A nonlinear association was observed between pre-pregnancy BMI and PTB (*P* for nonlinearity: <0.001). The risk of PTB increases at both low and high BMI levels, with the lowest risk of PTB occurring at approximately 21.6 kg/m^2^ [95% confidence interval (CI): (21.3–21.9)]. Subgroup analysis based on with or without previous termination or fetal loss revealed that the lowest risks of PTB were 22.6 kg/m^2^ (95% CI: 22.3–22.8) and 21.2 kg/m^2^ (95% CI: 20.8–21.6), respectively. The results of the sensitivity analyses remained stable.

**Conclusion:**

A nonlinear association between pre-pregnancy BMI and PTB was observed in women treated with ART. Lower and higher BMIs were associated with an increased risk of PTB, respectively, with the optimal pre-pregnancy BMI associated with the lowest risk of PTB being approximately 21.6 kg/m^2^. The lowest point may vary depending on whether there was a previous termination or fetal loss.

## Introduction

Preterm birth (PTB) (defined as delivery at less than 37 weeks of gestation) is the leading cause of death and long-term complications in children under 5 years of age (1), and its occurrence is strongly associated with inflammation, multiple pregnancies, reproductive history, malnutrition, and environmental and socioeconomic factors (2, 3). According to statistics, in 2019, 17.7% of deaths among children under the age of 5 worldwide (approximately 0.94 million) were caused by PTB (4). Although the mortality rate has decreased in recent years, PTB is still the leading cause (5). Surviving preterm infants are at higher risk for chronic conditions such as cerebral palsy, visual/hearing impairments, respiratory problems, and developmental delays (6). PTB is associated with an increased risk of mortality in adulthood, thereby triggering lifelong healthcare needs and economic burdens for individua ls (7).

Pre-pregnancy body mass index (BMI) is a key indicator for assessing baseline maternal nutritional status and obesity and is a recognized predictor of poor perinatal outcomes. The global prevalence of age-standardized underweight in women is estimated to decrease from 13.7% to 6.2%, and the prevalence of obesity is estimated to increase from 8.8% to 18.5% from 1990 to 2022 (8). The prevalence of obesity among adult women in the United States (U.S.) is also increasing each year, with an estimated percentage change of 47.9% from 1990 to 2021, a growth rate of 99.9%, and a projected prevalence of overweight and obesity of up to 82.1% by 2050 (9).

Previous studies have shown that both underweight (BMI <18.5 kg/m^2^) and obesity (BMI ≥ 30 kg/m^2^) are associated with an increased risk of PTB in natural pregnancies (10). The BMI distribution characteristics of assisted reproductive technology (ART) patients may differ from those of women who conceive naturally. The pre-pregnancy BMI distribution of this population may exhibit skewed characteristics due to factors such as metabolic abnormalities and polycystic ovary syndrome (PCOS), increasing the complexity of pregnancy management (11, 12). In addition, the ART technique itself may have a modifying effect on the BMI-PTB relationship due to endometrial factors (13, 14) and luteal function (15, 16). Previous national and international studies have suggested that ART pregnancies have a significantly higher risk of PTB than natural pregnancies (17–20) and are often associated with complications such as preeclampsia and gestational diabetes mellitus (20, 21). Therefore, independent analyses of this population are important for optimizing preconception management and improving pregnancy outcomes.

Currently, most studies of the association between pre-pregnancy BMI and PTB have focused on natural pregnancy populations and have not clearly differentiated between natural pregnancies and ART (10, 22), and the reasonableness of extrapolating their conclusions to ART populations is questionable. In summary, this study will delve into whether there is a nonlinear association between pre-pregnancy BMI and PTB in women who conceived through ART treatment based on a nationwide retrospective cohort study to determine the optimal pre-pregnancy BMI associated with the lowest risk of PTB. The aim is to provide a more precise reference for pre-pregnancy weight management and pregnancy healthcare for this group of women in order to improve the pregnancy outcome, reduce the PTB rate, and improve the perinatal health of the perinatal population.

## Materials and methods

### Database

This study used the publicly available National Vital Statistics System (NVSS) dataset (2019.01.01–2023.12.31) from the United States National Center for Health Statistics (NHCS), which contains detailed information on all births registered in the U.S. (23). The dataset is de-identified and publicly available to the public, and NCHS has assumed responsibility for obtaining ethical approval for data collection and publication. Therefore, no further ethical approval was required for this study. All analyses conform to the Statement for Reporting of Observational Studies in Epidemiology (STROBE) and follow the Vital Statistics User Agreement.

### Study population

The birth dataset from 2019 to 2023 (*n* = 18,328,446) was used in this study, and the following exclusion criteria were set: (i) women who conceived without ART (*n* = 18,045,994). ART includes *in vitro* fertilization (IVF) and gamete intrafallopian transfer (GIFT); (ii) multiple pregnancies (*n* = 42,015); (iii) non-live births (*n* = 1,173); (iv) age at delivery less than 18 years old (*n* = 8); (v) pre-pregnancy BMI information was missing (*n* = 3,439); (vi) gestational age <24 weeks or ≥42 weeks or missing (*n* = 758); (vii) missing data (*n* = 37,822).

### Exposure variable

The exposure variable in this study was pre-pregnancy BMI. Mother's pre-pregnancy BMI (kg/m^2^) is calculated as: (mother's pre-pregnancy weight (lb)/[mother's height (in)]^2^) × 703. Mother's height and pre-pregnancy weight information was collected through Mother's Worksheet. Pre-pregnancy BMI was used in continuous form in nonlinear relationship analyses. For descriptive and logistic regression analyses, pre-pregnancy BMI was categorized into 6 groups according to World Health Organization (WHO) criteria (24–26), including underweight (<18.50 kg/m^2^); normal weight (18.5–24.9 kg/m^2^); overweight (25–29.9 kg/m^2^) and obesity (Class I: 30–34.9 kg/m^2^, Class II: 35–39.9 kg/m^2^ and Class III: ≥ 40 kg/m^2^), of which 18.5–24.9 kg/m^2^ was the reference group.

### Outcomes

The primary outcome was PTB (<37 weeks), and the secondary outcomes were the categories of PTB. The World Health Organization (WHO) defines preterm birth as delivery occurring before 37 weeks of gestation. PTB is divided into 3 subtypes according to the number of weeks of gestation: extremely preterm (<28 weeks), very preterm (28 to <32 weeks), and moderate to late preterm (32 to <37 weeks) (1). Gestational weeks data integrated obstetric estimates (OE) using data from ultrasound, last menstrual period (LMP), and physical examination. The specific methods of assessment were described in detail in our previous study (27). Researchers have shown that the method of estimating PTB rates based on OE has been shown to have excellent specificity and negative and positive predictive value (28).

### Covariates

Collection of information on relevant potential confounders: age of the pregnancy (year) (age at delivery minus gestational age), race (White, Black, Asian, or other), marital status (married or unmarried), education level (high school and below or higher than high school), smoking before pregnancy (yes or no), insurance (Medicaid, private, self-pay, or other), parity before the current pregnancy (0, or ≥ 1), obstetrical history (previous termination or fetal loss, neonatal death, PTB, or cesarean section), and pre-pregnancy comorbidities (pre-pregnancy diabetes or hypertension).

### Missing data

Except for marital status (14.72%), the incidence of missing data for other covariates was low (<4%; Supplementary Table 1). This was mainly due to California's systematic cessation of providing information on mothers' marital status since 2017 due to state law restrictions (29–31). The absence of marital status information is directly related to the privacy of marital status records themselves (such as “married/unmarried”). Little's missing completely at random (MCAR) test rejected the assumption of MCAR (*P* < 0.001, Supplementary Table 2), suggesting that data missingness may be either missing at random (MAR) or missing not at random (MNAR). Based on background information and relevance analysis (Supplementary material 2), the missingness may depend on observed variables (such as maternal age, race, and education level) and marital status itself (i.e., marital status information involves privacy concerns, and California law prohibits its reporting). Given the complexity and uncertainty surrounding missing data mechanisms, we selected complete case analysis (CCA) as a conservative approach to minimize potential bias. CCA has been demonstrated to yield valid results when missing data are MAR or close to MNAR, particularly when the proportion of missing data is relatively low (32, 33).

### Statistical analysis

Continuous variables are presented as mean ± standard deviation (SD), and categorical variables are presented as frequencies or percentages. For analysis of baseline characteristics, statistical differences between pre-pregnancy BMI groups were tested using *t*-tests or one-way analysis of variance (ANOVA) for continuous variables and chi-squared or Fisher tests for categorical variables.

We examined the relationship between pre-pregnancy BMI and PTB in individuals who conceived after ART treatment using logistic regression models. The following covariates were used to adjust the multivariable model: age of the pregnancy, race, marital status, education level, smoking before pregnancy, insurance, parity before the current pregnancy, previous termination or fetal loss, previous neonatal death, previous PTB, previous cesarean section, pre-pregnancy diabetes, and pre-pregnancy hypertension to calculate adjusted odds ratios (ORs) and 95% confidence intervals (95% CIs). The selection criteria for the inclusion of covariates were determined by an integrated approach that considered univariate regression analysis (Supplementary Table 3), reports in the relevant literature, and a combination of Directed Acyclic Graphs (DAGs) guidance (Supplementary Figure 1).

To further explore the potential nonlinear dose-response relationship between pre-pregnancy BMI and PTB, we developed a restricted cubic spline (RCS) model with 4 nodes (5^th^, 35^th^, 65^th^, and 95^th^). The likelihood ratio test was used for non-linearity hypothesis testing (*P* for non-linearity). The bootstrap resampling method and likelihood ratio test were employed to determine the minimum point and its confidence interval. In this model, pre-pregnancy BMI was used as a continuous variable, and subjects within the mean ± 3SD range (*n* = 195,180) were included. Using the median as a reference point, the corresponding odds ratio and 95% confidence interval (CI) were calculated after adjusting for all covariates. The results were presented graphically to intuitively show the relationship between changes in BMI levels and the risk of PTB.

Subgroup analyses were performed based on with or without previous termination or fetal loss, and interactions were assessed using likelihood ratio tests.

Pre-pregnancy diabetes and pre-pregnancy hypertension may have causal mechanisms (mediating pathways) that influence PTB through BMI, rather than simply being confounding factors (34–37). These two conditions have dual attributes in the analysis: they may be confounding factors that need to be adjusted for, or they may be mediating variables in the association between BMI and PTB. Therefore, sensitivity analysis excluded not only women with a history of PTB but also participants with pre-pregnancy diabetes and pre-pregnancy hypertension in order to assess confounding effects and the robustness of the association. In addition, we adjusted the analysis range for pre-pregnancy BMI.

All analyses were performed using R Statistical Software (Version 4.2.2, http://www.R-project.org, The R Foundation) and the Free Statistics analysis platform (Version 2.1.1, Beijing, China). Statistical testing was two-sided. Statistical significance was defined as *P* < 0.05.

## Results

### Baseline characteristics

**Figure 1:**
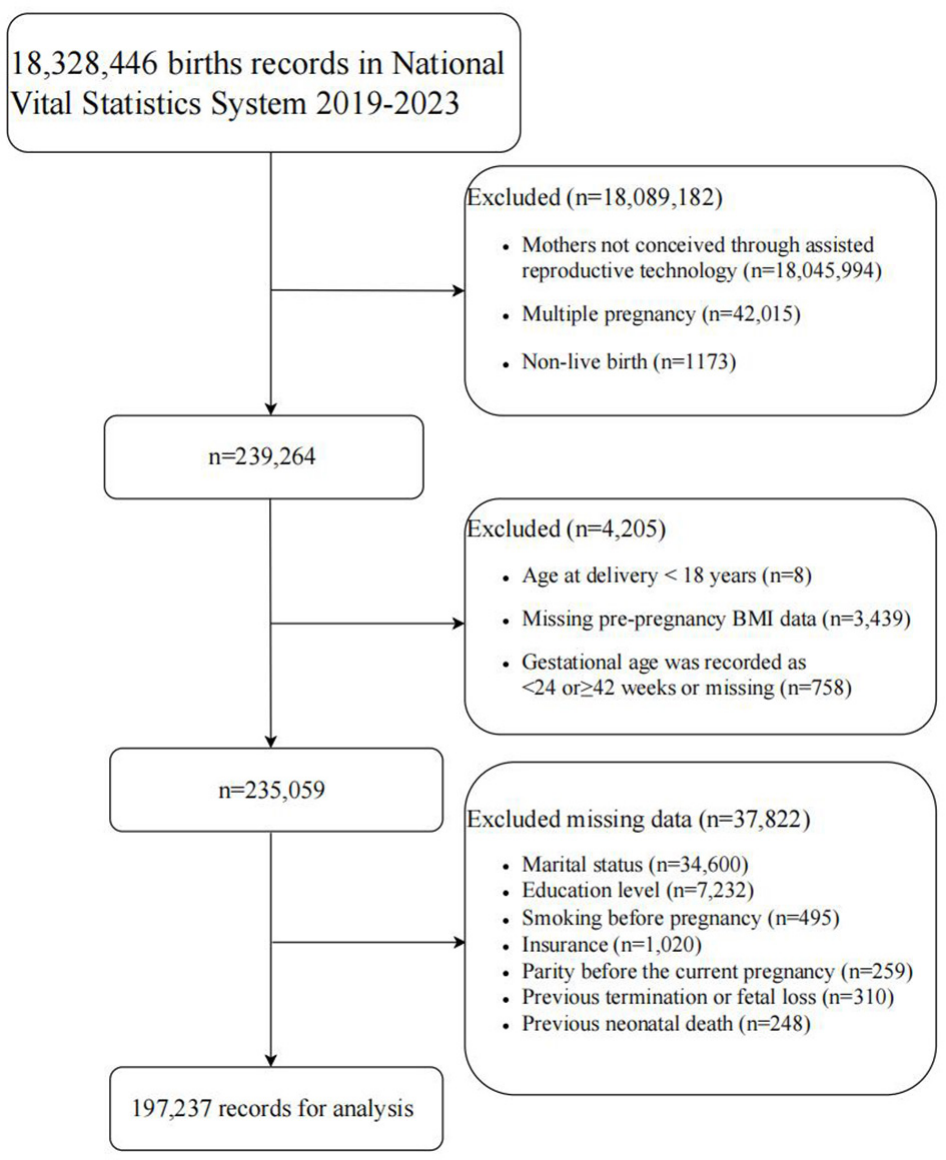
Flowchart of participant selection. Multiple pieces of covariate information may be missing for the same research subject. BMI, body mass index (calculated as weight in kilograms divided by the square of height in meters).

A total of 18,328,446 records were included in the NVSS 2019–2023 birth dataset. Based on the exclusion criteria, 197,237 cases were ultimately included in the analysis (Figure 1). The mean age of the mothers participating in the study was 35 ± 5 years; 79.68% were White; the majority (*n* = 89,603,45.43%) had a pre-pregnancy BMI of 18.5–24.9 kg/m^2^, 3,643 (1.85%) were <18.50 kg/m^2^, 53,248 (27.00%) were 25–29.9 kg/m^2^, 28,686 (14.54%) were 30–34.9 kg/m^2^, 14,195 (7.20%) were 35–39.9 kg/m^2^, and 7,862 (3.99%) were ≥40 kg/m^2^; 22,592 (11.45%) were PTB, and the group of 18.5–24.9 kg/m^2^ had the lowest rate of PTB (9.09%). Detailed baseline characteristics are shown in Table 1.

**Table 1:** Population characteristics by categories of pre-pregnancy BMI.

**Characteristic**	**Pre-pregnancy BMI**							***p*-value**
	**Total (*****n*** = **197,237)**	<**18.5 (*****n*** = **3,643)**	**18.5–24.9 (*****n*** = **89,603)**	**25.0–29.9 (*****n*** = **53,248)**	**30.0–34.9 (*****n*** = **28,686)**	**35.0–39.9 (*****n*** = **14,195)**	≥**40.0 (*****n*** = **7,862)**	
Age of the pregnancy (year), mean ± SD	35 ± 5	34 ± 5	35 ± 5	35 ± 5	35 ± 5	34 ± 5	34 ± 5	<0.001
Race, *n* (%)								<0.001
White	157,154 (79.68)	2,564 (70.38)	71,672 (79.99)	41,845 (78.59)	22,757 (79.33)	11,621 (81.87)	6,695 (85.16)	
Black	12,692 (6.43)	107 (2.94)	3,136 (3.5)	4,139 (7.77)	2,939 (10.25)	1,567 (11.04)	804 (10.23)	
Asian	23,135 (11.73)	904 (24.81)	13,059 (14.57)	6,086 (11.43)	2,274 (7.93)	652 (4.59)	160 (2.04)	
Other	4,256 (2.16)	68 (1.87)	1,736 (1.94)	1,178 (2.21)	716 (2.5)	355 (2.5)	203 (2.58)	
Marital status, *n* (%)								<0.001
Married	179,681 (91.10)	3,386 (92.95)	83,124 (92.77)	48,092 (90.32)	25,545 (89.05)	12,571 (88.56)	6,963 (88.57)	
Unmarried	17,556 (8.90)	257 (7.05)	6,479 (7.23)	5,156 (9.68)	3,141 (10.95)	1,624 (11.44)	899 (11.43)	
Education level, *n* (%)								<0.001
High school and below	13,333 (6.76)	255 (7)	4,401 (4.91)	3,922 (7.37)	2,636 (9.19)	1,317 (9.28)	802 (10.2)	
Higher than high school	183,904 (93.24)	3,388 (93)	85,202 (95.09)	49,326 (92.63)	26,050 (90.81)	12,878 (90.72)	7,060 (89.8)	
Smoking before pregnancy, *n* (%)								<0.001
No	195,801 (99.27)	3,623 (99.45)	89,203 (99.55)	52,816 (99.19)	28,397 (98.99)	14,009 (98.69)	7,753 (98.61)	
Yes	1,436 (0.73)	20 (0.55)	400 (0.45)	432 (0.81)	289 (1.01)	186 (1.31)	109 (1.39)	
Insurance, *n* (%)								<0.001
Medicaid	9,859 (5.00)	203 (5.57)	3,405 (3.8)	2,969 (5.58)	1,857 (6.47)	921 (6.49)	504 (6.41)	
Private insurance	180,502 (91.52)	3,318 (91.08)	83,115 (92.76)	48,296 (90.7)	25,740 (89.73)	12,849 (90.52)	7,184 (91.38)	
Self-pay	2,261 (1.15)	59 (1.62)	1,082 (1.21)	628 (1.18)	335 (1.17)	99 (0.7)	58 (0.74)	
Other	4,615 (2.34)	63 (1.73)	2,001 (2.23)	1,355 (2.54)	754 (2.63)	326 (2.3)	116 (1.48)	
Parity before the current pregnancy, *n* (%)								<0.001
0	115,320 (58.47)	2,225 (61.08)	52,484 (58.57)	30,294 (56.89)	16,538 (57.65)	8,802 (62.01)	4,977 (63.3)	
≥1	81,917 (41.53)	1,418 (38.92)	37,119 (41.43)	22,954 (43.11)	12,148 (42.35)	5,393 (37.99)	2,885 (36.7)	
Previous termination or fetal loss, *n* (%)								<0.001
No	120,553 (61.12)	2,383 (65.41)	55,940 (62.43)	32,332 (60.72)	17,008 (59.29)	8,337 (58.73)	4,553 (57.91)	
Yes	76,684 (38.88)	1,260 (34.59)	33,663 (37.57)	20,916 (39.28)	11,678 (40.71)	5,858 (41.27)	3,309 (42.09)	
Previous neonatal death, *n* (%)								<0.001
No	195,264 (99.00)	3,613 (99.18)	88,874 (99.19)	52,681 (98.94)	28,327 (98.75)	14,005 (98.66)	7,764 (98.75)	
Yes	1,973 (1.00)	30 (0.82)	729 (0.81)	567 (1.06)	359 (1.25)	190 (1.34)	98 (1.25)	
Previous preterm birth, *n* (%)								<0.001
No	190,222 (96.44)	3,535 (97.04)	86,818 (96.89)	51,285 (96.31)	27,456 (95.71)	13,611 (95.89)	7,517 (95.61)	
Yes	7,015 (3.56)	108 (2.96)	2,785 (3.11)	1,963 (3.69)	1,230 (4.29)	584 (4.11)	345 (4.39)	
Previous cesarean section, *n* (%)								<0.001
No	166,703 (84.52)	3,277 (89.95)	77,254 (86.22)	44,545 (83.66)	23,543 (82.07)	11,684 (82.31)	6,400 (81.4)	
Yes	30,534 (15.48)	366 (10.05)	12,349 (13.78)	8,703 (16.34)	5,143 (17.93)	2,511 (17.69)	1,462 (18.6)	
Pre-pregnancy diabetes, *n* (%)								<0.001
No	194,580 (98.65)	3,628 (99.59)	89,121 (99.46)	52,615 (98.81)	28,056 (97.8)	13,672 (96.32)	7,488 (95.24)	
Yes	2,657 (1.35)	15 (0.41)	482 (0.54)	633 (1.19)	630 (2.2)	523 (3.68)	374 (4.76)	
Pre-pregnancy hypertension, *n* (%)								<0.001
No	188,533 (95.59)	3,616 (99.26)	88,190 (98.42)	51,172 (96.1)	26,551 (92.56)	12,501 (88.07)	6,503 (82.71)	
Yes	8,704 (4.41)	27 (0.74)	1,413 (1.58)	2,076 (3.9)	2,135 (7.44)	1,694 (11.93)	1,359 (17.29)	
Preterm birth, *n* (%)								<0.001
Yes	22,592 (11.45)	369 (10.13)	8,149 (9.09)	6,238 (11.71)	4,130 (14.4)	2,305 (16.24)	1,401 (17.82)	
<28 weeks	1,143 (0.58)	17 (0.47)	297 (0.33)	341 (0.64)	256 (0.89)	147 (1.04)	85 (1.08)	
28 to <32 weeks	2,044 (1.04)	27 (0.74)	677 (0.76)	551 (1.03)	410 (1.43)	236 (1.66)	143 (1.82)	
32 to <37 weeks	19,405 (9.84)	325 (8.92)	7,175 (8.01)	5,346 (10.04)	3,464 (12.08)	1,922 (13.54)	1,173 (14.92)	
No	174,645 (88.55)	3,274 (89.87)	81,454 (90.91)	47,010 (88.29)	24,556 (85.6)	11,890 (83.76)	6,461 (82.18)	

BMI, body mass index (calculated as weight in kilograms divided by the square of height in meters); SD, standard deviation.

### Nonlinear association between pre-pregnancy BMI and PTB

**Figure 2:**
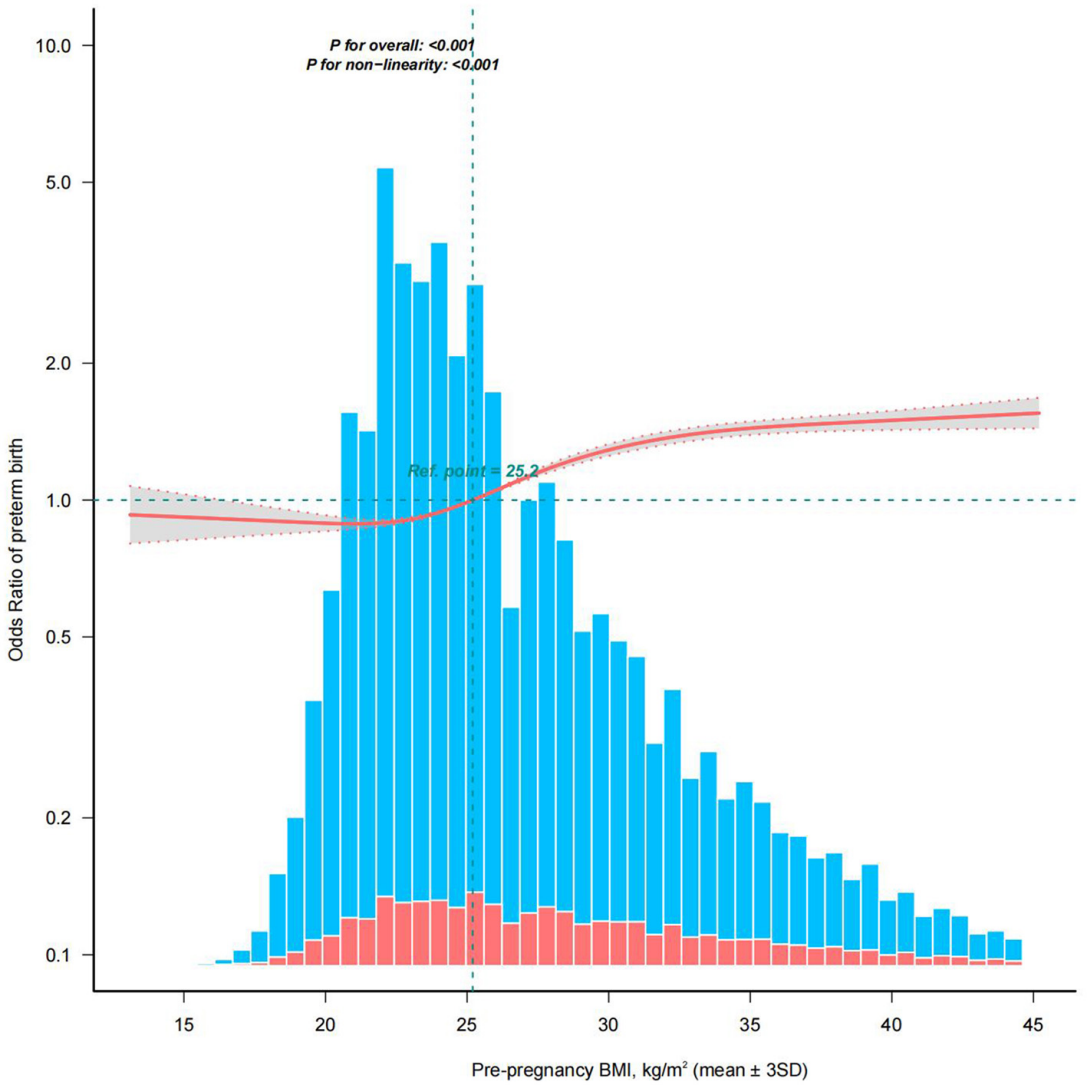
Restricted cubic spline model of the relationship between pre-pregnancy BMI and PTB. The solid red line and the gray area represent the ORs and their corresponding 95% CIs, respectively. The orange and blue bars represent the pre-pregnancy BMI distribution of people with and without a PTB, respectively. Adjusted for age of the pregnancy, race, marital status, education level, smoking before pregnancy, insurance, parity before the current pregnancy, previous termination or fetal loss, previous neonatal death, previous PTB, previous cesarean section, pre-pregnancy diabetes, and pre-pregnancy hypertension. Only data for the pre-pregnancy BMI in the range of mean ± 3SD (*n* = 195,180) are shown. BMI, body mass index; PTB, preterm birth; SD, standard deviation; OR, odds ratio; CI, confidence interval.

The association between pre-pregnancy BMI and PTB is shown in Table 2, including crude models without adjustment for covariates and adjusted models after adjustment for all covariates. Compared with the reference group, pre-pregnancy BMI <18.5 and ≥ 25 kg/m^2^ were both associated with an increased risk of PTB. In the adjusted model OR (95% CI) for BMI <18.5 kg/m^2^: 1.14 (1.02–1.27); 25–29.9 kg/m^2^: 1.26 (1.21–1.30); 30–34.9 kg/m^2^: 1.50 (1.44–1.56); 35–39.9 kg/m^2^: 1.64 (1.55–1.72); ≥40 kg/m^2^: 1.75 (1.64–1.87). Subsequently, in the RCS model analysis adjusted for all covariates, participants with pre-pregnancy BMI within the mean ± 3SD range (*n* = 195,180) were included. A nonlinear relationship was observed between pre-pregnancy BMI and PTB (*P* for non-linearity: <0.001, Figure 2), with the lowest risk of PTB at approximately 21.6 (21.3, 21.9), followed by a gradual increase in risk, which then plateaued after reaching 35.7 (35.5, 35.9).

**Table 2:** The logistic regression of pre-pregnancy BMI associated with PTB.

**Pre-pregnancy BMI**	***n*. Total**	***n*. Event %**	**Crude OR (95%CI)**	**Crude *p*-value**	**Adjusted OR (95% CI)**	**Adjusted *p*-value**
<18.5	3,643	369 (10.1)	1.13 (1.01–1.26)	0.034	1.14 (1.02–1.27)	0.021
18.5–24.9	89,603	8,149 (9.1)	1 (Ref)		1 (Ref)	
25.0–29.9	53,248	6,238 (11.7)	1.33 (1.28–1.37)	<0.001	1.26 (1.21–1.30)	<0.001
30.0–34.9	28,686	4,130 (14.4)	1.68 (1.62–1.75)	<0.001	1.50 (1.44–1.56)	<0.001
35.0–39.9	14,195	2,305 (16.2)	1.94 (1.84–2.04)	<0.001	1.64 (1.55–1.72)	<0.001
≥40.0	7,862	1,401 (17.8)	2.17 (2.04–2.31)	<0.001	1.75 (1.64–1.87)	<0.001

Adjusted for age of the pregnancy, race, marital status, education level, smoking before pregnancy, insurance, parity before the current pregnancy, previous termination or fetal loss, previous neonatal death, previous PTB, previous cesarean section, pre-pregnancy diabetes, and pre-pregnancy hypertension. BMI, body mass index; PTB, preterm birth; OR, odds ratio; CI, confidence interval; Ref, reference.

In the multinomial logistic regression model for different subgroups of PTB (Supplementary Table 4), similar results were observed in the moderate to late PTB group, while no statistically significant differences were found in the very PTB and extremely PTB groups when maternal BMI was <18.5 kg/m^2^ before pregnancy [0.97 (0.66–1.42); 1.34 (0.82–2.18)]. The trend with increasing BMI appears to be slightly stronger for very PTB and extremely PTB than in the moderate to late PTB group.

### Subgroup analysis

An interaction was observed in the subgroup analysis based on with or without previous termination or fetal loss (Supplementary Table 5; *P* for interaction = 0.002). In multivariate-adjusted logistic regression, pre-pregnancy BMI <18.5 kg/m^2^ was associated with an increased risk of PTB in the subgroup without previous termination or fetal loss [OR (95% CI): 1.18 (1.03–1.36), *P* = 0.015], while there was no statistically significant difference in PTB risk in the subgroup with previous termination or fetal loss [OR (95% CI): 1.06 (0.88–1.28), *P* = 0.549]. In the groups with pre-pregnancy BMI ≥ 25 kg/m^2^, it was observed that the risk of PTB increased with increasing BMI. This was more evident in subgroups without previous termination or fetal loss in the 25.0–29.9 kg/m^2^ and 30.0–34.9 kg/m^2^ groups and more evident in subgroups with previous termination or fetal loss in the 35.0–39.9 kg/m^2^ and ≥ 40.0 kg/m^2^ groups. In the multivariate-adjusted RCS model (Supplementary Figure 2), the lowest point of PTB risk in the subgroup with previous termination or fetal loss occurred at approximately 22.6 (22.3, 22.8), while in the subgroup without previous termination or fetal loss, it occurred at approximately 21.2 (20.7, 21.6). The two CI groups did not overlap, further supporting the modifying effect of previous termination or fetal loss on the association between BMI and PTB.

### Sensitivity analysis

**Figure 3:**
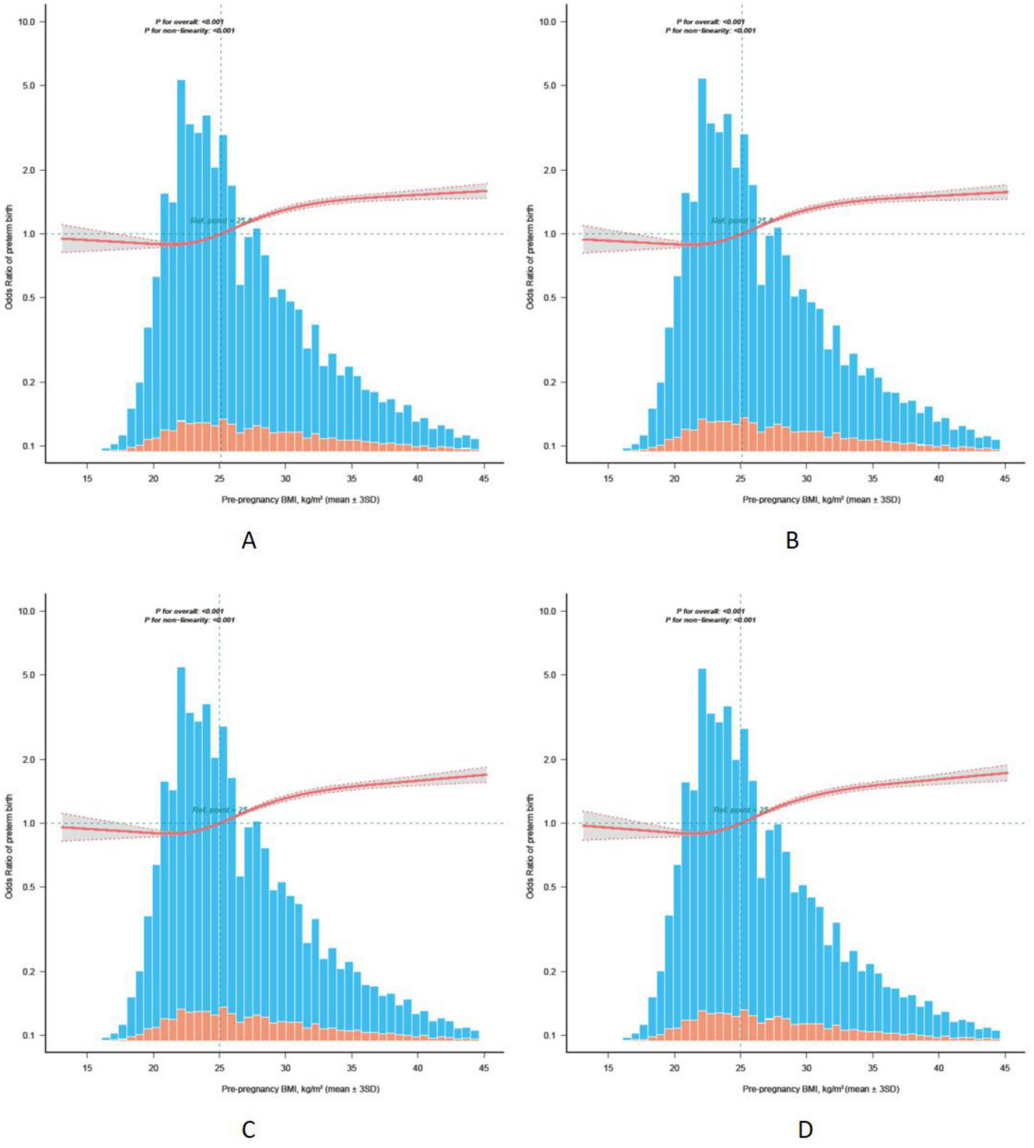
Sensitivity analysis of the non-linear association between pre-pregnancy BMI and PTB. **(A)** Subjects without previous preterm birth (*n* = 188,248). **(B)** Subjects without pre-pregnancy diabetes (*n* = 192,632). **(C)** Subjects without pre-pregnancy hypertension (*n* = 186,927). **(D)** Subjects without previous preterm birth, pre-pregnancy diabetes, and pre-pregnancy hypertension (*n* = 178,669). The solid red line and the gray area represent the ORs and their corresponding 95% CIs, respectively. The orange and blue bars represent the pre-pregnancy BMI distribution of people with and without a PTB, respectively. Age of the pregnancy, race, marital status, education level, smoking before pregnancy, insurance, parity before the current pregnancy, previous termination or fetal loss, previous neonatal death, previous preterm birth, previous cesarean section, pre-pregnancy diabetes, and pre-pregnancy hypertension were adjusted for in the model except when the variable was excluded. BMI, body mass index; PTB, *p*reterm birth; SD, standard deviation; OR, odds ratio; CI, confidence interval.

The results of the sensitivity analysis (Figure 3, Table 3, Supplementary Table 6) were similar to the overall results, with no evidence of a change in the nonlinear association between pre-pregnancy BMI and PTB in women who conceived with ART treatment.

**Table 3:** Sensitivity analysis for the association of pre-pregnancy BMI with PTB.

**Variable**	***n*. Total**	***n*. Event %**	**Crude OR (95%CI)**	**Crude *p*-value**	**Adjusted OR (95% CI)**	**Adjusted *p*-value**
**Subjects without previous preterm birth (*****n*** = **190,222)**						
<18.5	3,535	341 (9.6)	1.13 (1.01–1.26)	0.039	1.14 (1.01–1.27)	0.029
18.5–24.9	86,818	7,510 (8.7)	1(Ref)		1(Ref)	
25.0–29.9	51,285	5,704 (11.1)	1.32 (1.27–1.37)	<0.001	1.26 (1.21–1.31)	<0.001
30.0–34.9	27,456	3,739 (13.6)	1.66 (1.6–1.74)	<0.001	1.51 (1.45–1.58)	<0.001
35.0–39.9	13,611	2,110 (15.5)	1.94 (1.84–2.04)	<0.001	1.66 (1.57–1.75)	<0.001
≥40.0	7,517	1,284 (17.1)	2.18 (2.04–2.32)	<0.001	1.78 (1.66–1.90)	<0.001
**Subjects without pre-pregnancy diabetes (*****n*** = **194,580)**						
<18.5	3,628	368 (10.1)	1.14 (1.02–1.27)	0.022	1.15 (1.03–1.28)	0.015
18.5–24.9	89,121	8,045 (9.0)	1(Ref)		1(Ref)	
25.0–29.9	52,615	6,081 (11.6)	1.32 (1.27–1.36)	<0.001	1.26 (1.21–1.30)	<0.001
30.0–34.9	28,056	3,963 (14.1)	1.66 (1.59–1.73)	<0.001	1.51 (1.45–1.57)	<0.001
35.0–39.9	13,672	2,143 (15.7)	1.87 (1.78–1.97)	<0.001	1.64 (1.55–1.73)	<0.001
≥40.0	7,488	1,291 (17.2)	2.1 (1.97–2.24)	<0.001	1.78 (1.66–1.90)	<0.001
**Subjects without pre-pregnancy hypertension (*****n*** = **188,533)**						
<18.5	3,616	365 (10.1)	1.15 (1.03–1.29)	0.012	1.16 (1.03–1.29)	0.01
18.5–24.9	88,190	7,829 (8.9)	1 (Ref)		1(Ref)	
25.0–29.9	51,172	5,766 (11.3)	1.3 (1.26–1.35)	<0.001	1.26 (1.22–1.31)	<0.001
30.0–34.9	26,551	3,578 (13.5)	1.6 (1.53–1.67)	<0.001	1.51 (1.45–1.58)	<0.001
35.0–39.9	12,501	1,880 (15.0)	1.82 (1.72–1.92)	<0.001	1.69 (1.60–1.79)	<0.001
≥40.0	6,503	1,083 (16.7)	2.05 (1.91–2.2)	<0.001	1.90 (1.77–2.04)	<0.001
**Subjects without previous preterm birth, pre-pregnancy diabetes, pre-pregnancy hypertension (*****n*** = **180,154)**						
<18.5	3,497	338 (9.7)	1.17 (1.04–1.31)	0.008	1.16 (1.04–1.31)	0.01
18.5–24.9	85,053	7,141 (8.4)	1(Ref)		1(Ref)	
25.0–29.9	48,839	5,178 (10.6)	1.29 (1.25–1.34)	<0.001	1.27 (1.22–1.32)	<0.001
30.0–34.9	25,045	3,157 (12.6)	1.57 (1.51–1.65)	<0.001	1.52 (1.46–1.60)	<0.001
35.0–39.9	11,691	1,645 (14.1)	1.79 (1.69–1.89)	<0.001	1.72 (1.62–1.82)	<0.001
≥40.0	6,029	943 (15.6)	2.02 (1.88–2.18)	<0.001	1.95 (1.81–2.10)	<0.001
**Subjects with a pre-pregnancy BMI of mean** ±**3SD (*****n*** = **195,180)**						
<18.5	3,643	369 (10.1)	1.13 (1.01–1.26)	0.034	1.14 (1.02–1.27)	0.021
18.5–24.9	89,603	8,149 (9.1)	1(Ref)		1(Ref)	
25.0–29.9	53,248	6,238 (11.7)	1.33 (1.28–1.37)	<0.001	1.25 (1.21–1.30)	<0.001
30.0–34.9	28,686	4,130 (14.4)	1.68 (1.62–1.75)	<0.001	1.50 (1.44–1.56)	<0.001
35.0–39.9	14,195	2,305 (16.2)	1.94 (1.84–2.04)	<0.001	1.63 (1.55–1.72)	<0.001
≥40.0	5,805	1,014 (17.5)	2.12 (1.97–2.27)	<0.001	1.73 (1.60–1.86)	<0.001

Age of the pregnancy, race, marital status, education level, smoking before pregnancy, insurance, parity before the current pregnancy, previous termination or fetal loss, previous neonatal death, previous preterm birth, previous cesarean section, pre-pregnancy diabetes, and pre-pregnancy hypertension were adjusted for in the model except when the variable was excluded. BMI, body mass index; PTB, preterm birth; OR, odds ratio; CI, confidence interval; Ref, reference.

## Discussion

In this nationwide retrospective cohort study, we sought to analyze the correlation between pre-pregnancy BMI and the risk of PTB in women who conceived through ART, as well as the lowest point of risk. A nonlinear association between pre-pregnancy BMI and risk of PTB was found in this cohort. The optimal pre-pregnancy BMI associated with the lowest risk of PTB is approximately 21.6 (21.3, 21.9) kg/m^2^.

Our study found that there is a nonlinear association between pre-pregnancy BMI and PTB risk in women who conceived through ART, with a minimum point of 21.6 kg/m^2^ (21.3, 21.9). This value is highly similar to the results of studies on naturally pregnant populations. Previous studies have generally supported a “J-shaped” or “V-shaped” curve relationship between pre-pregnancy BMI and PTB (10, 38, 39), with the lowest risk point mostly in the upper-middle range of normal BMI (22.5–25.9 kg/m^2^). These slight differences may be related to differences in metabolic characteristics among women undergoing ART. ART patients are often comorbid with underlying conditions such as polycystic ovary syndrome (PCOS) and endometriosis, and their endocrine milieu and high-estrogenic state due to ovulation induction therapy may alter the threshold for the BMI-PTB association (40–43). Existing ART-related studies have mostly focused on single centers or regional data (44–46), with limited sample sizes and a lack of national representation. This study incorporated diverse covariates, such as race, age, and reproductive history, based on the large data strengths of the NVSS (*n* = 197,237), to further validate the presence of a nonlinear association and capture nonlinear features more precisely through the RCS model. The study also refined the lowest point. The nonlinear association of pre-pregnancy BMI with PTB may stem from multifaceted pathophysiologic mechanisms. Low-weight women may have limited placental trophoblast invasion in early pregnancy due to malnutrition and inadequate fat reserves, which in turn affects uteroplacental perfusion (47, 48). In addition, low BMI is associated with low levels of leptin, which plays a key role in regulating the hypothalamic-pituitary-ovarian axis and maintaining luteal function in early pregnancy (49, 50). In contrast, the chronic low-grade inflammatory state (e.g., elevated C-reactive protein and interleukin-6) in overweight/obese women induces oxidative stress in ovarian and uterine metamorphic tissues (51–53), promoting prostaglandin synthesis and premature cervical ripening. Meanwhile, high BMI is closely associated with insulin resistance and disorders of lipid metabolism (54, 55), which may cause histopathological changes in the placenta leading to the occurrence of PTB (56, 57). It is worth noting that the high proportion of overweight and obese women in the ART population may cause metabolic stress in women with superovulation, leading to abnormal follicular microenvironments and decreased embryo quality (58), while higher levels of pathogenic bacteria in the uterine flora of overweight or obese women may also impair endometrial tolerance, ultimately leading to adverse pregnancy outcomes (59).

This study is based on the NVSS's national data coverage of U.S. birth registrations, which effectively reduces selection bias and has stronger extrapolation, especially for minority populations such as ART. The RCS model was used to fit the BMI-PTB curve, breaking away from traditional linear assumptions, identifying threshold effects more accurately, and controlling for confounders through multivariate adjustment (including age, race, smoking, and reproductive history). This study focuses on the ART population, a high-risk group, to provide specific evidence for pre-pregnancy BMI management. Additionally, this study is founded on clinically accessible base information, thereby enhancing the generalizability of the results. Nevertheless, this study still has a few limitations. First, the NVSS data lacks details on hormone levels in early pregnancy, ART-specific protocols [e.g., frozen-thawed embryo transfer (FET) vs. fresh embryo transfer (ET)], and embryo quality and may omit key confounding variables. Second, pre-pregnancy BMI is based on self-reporting, which may lead to classification errors due to recall bias, especially in extreme BMI populations (e.g., emaciated or obese individuals); In the multivariate logistic regression of PTB, there was no statistical difference between very PTB and extremely PTB groups in pre-pregnancy BMI <18.5 kg/m^2^ [0.97 (0.66–1.42); 1.34 (0.82–2.18)]. This seems to be related to the extreme reduction of sample size [27 (0.7); 17 (0.5)]. A larger sample size study is needed to verify the subtype-specific association. Third, due to the lack of relevant data, lifestyle (e.g., dietary patterns, exercise) and psychosocial factors (e.g., stress during pregnancy) were not included. To make sure the results were reliable, several subgroup analyses and sensitivity analyses were performed. The results of sensitivity analysis were similar to the main results. Notably, in the subgroup with previous termination or fetal loss, the 95% CI for pre-pregnancy BMI <18.5 kg/m^2^ included 1.0 [1.06 (0.88–1.28), *P* = 0.549], indicating that the association between BMI <18.5 kg/m^2^ and PTB risk was statistically insignificant in this subgroup (i.e., there is little evidence suggesting an association). This may also be related to the relatively small sample size of the subgroup (*n* = 126, 10.0%). In addition, as an observational study, the causal relationship between preconception BMI and PTB risk in those conceived via ART could not be determined and needs to be further explored and validated.

## Conclusion

A nonlinear association exists between pre-pregnancy BMI and PTB among women treated with ART in the U.S. Lower and higher BMIs are both associated with an increased risk of PTB. The optimal pre-pregnancy BMI associated with the lowest risk of PTB is approximately 21.6 kg/m^2^. This value may vary depending on whether there was a previous termination or fetal loss.

## Data Availability

The raw data supporting the conclusions of this article will be made available by the authors, without undue reservation.
