# Supplementary material for: Nonlinear association between pre-pregnancy body mass index and preterm birth in singleton pregnancies conceived with assisted reproductive technology
Source: Front Nutr. 2026 Jan 12;12:1651462. doi: 10.3389/fnut.2025.1651462 (PMC12833966; doi:10.3389/fnut.2025.1651462)
Supplement: Supplementary file 1 [file Table_1.docx]

**Supplement Content**

**Supplemental figure 1.** Directed acyclic graph (DAG) illustrating confounder selection.

**Supplemental figure 2.** Restricted cubic spline model of the relationship between pre-pregnancy BMI and PTB. (based on the with (A) or without (B) previous termination or fetal loss).

**Supplemental table 1.** The descriptive statistics for the missing covariates.

**Supplemental table 2.** Little’s MCAR test.

**Supplemental table 3.** Association of covariates and PTB.

**Supplemental table 4.** Multinomial logistic regression of pre-pregnancy BMI and PTB.

**Supplemental table 5.** Subgroup analyses between pre-pregnancy BMI and PTB by previous termination or fetal loss.

**Supplemental table 6.** Sensitivity analysis for multinomial logistic regression of pre-pregnancy BMI and PTB.


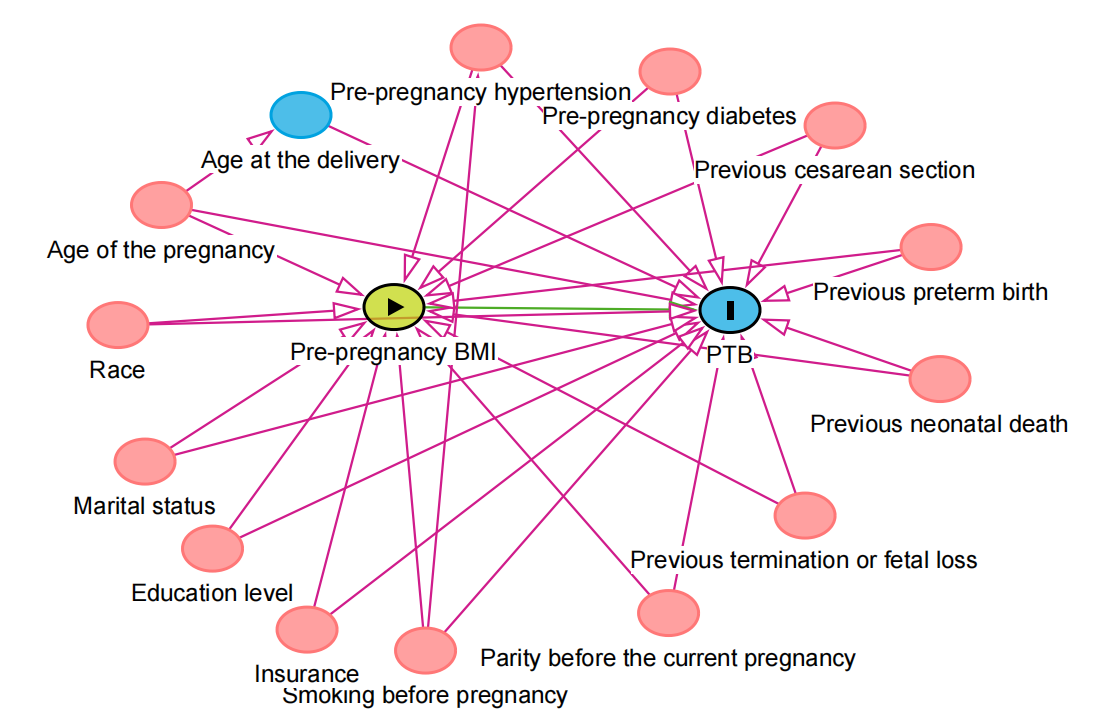


**Supplemental figure 1.** Directed acyclic graph (DAG) illustrating confounder selection.

**Abbreviations:** BMI, body mass index; PTB, preterm birth.

A B

**Supplemental figure 2.** Restricted cubic spline model of the relationship between pre-pregnancy BMI and PTB. (based on the with (A) or without (B) previous termination or fetal loss).

The solid red line and the gray area represent the ORs and their corresponding 95% CIs, respectively. The orange and blue bars represent the pre-pregnancy BMI distribution of people with and without a PTB, respectively.

Adjusted for age of the pregnancy, race, marital status, education level, smoking before pregnancy, insurance, parity before the current pregnancy, previous neonatal death, previous preterm birth, previous cesarean section, pre-pregnancy diabetes, and pre-pregnancy hypertension.

Only data for the pre-pregnancy BMI in the range of mean ± 3SD are shown (n=75,823 (A), n=119,357 (B)).

**Abbreviations:** BMI, body mass index; PTB, preterm birth; SD, standard deviation; OR, odds ratio; CI, confidence interval.

**Supplemental table 1.** **The descriptive statistics for the missing covariates.**

| Variable | Complete(n,%) | Missing(n,%) |
| --- | --- | --- |
| Mariral status | 200459(85.82) | 34600(14.72) |
| Education level | 227827(96.92) | 7232(3.08) |
| Smoking before pregnancy | 234564(99.79) | 495(0.21) |
| Insurance | 234039(99.57) | 1020(0.43) |
| Parity before the current pregnancy | 234800(99.89) | 259(0.11) |
| Previous termination | 234749(99.87) | 310(0.13) |
| Previous neonatal death | 234811(99.89) | 248(0.11) |
| Whole sample | 197237(83.91) | 37822(16.09) |

**Supplemental table 2. Little’s MCAR test.**

| chi.square | degree.free | p.value | missing.patterns |
| --- | --- | --- | --- |
| 22511.15459 | 504 | <0.001 | 39 |

**Supplemental table 3.** **Association of covariates and PTB.**

| Variable | OR（95%CI） | P-value |
| --- | --- | --- |
| Age of the pregnancy(years) | 1.02 (1.02~1.02) | <0.001 |
| Race, n (%) |  |  |
| White | 1(Ref) |  |
| Black | 1.65 (1.58~1.74) | <0.001 |
| Asian | 1.03 (0.98~1.07) | 0.228 |
| Other | 1.17 (1.07~1.29) | 0.001 |
| Marital status, n (%) |  |  |
| Married | 1(Ref) |  |
| Unmarried | 1.19 (1.14~1.25) | <0.001 |
| Education level, n (%) |  |  |
| High school and below | 1(Ref) |  |
| Higher than high school | 0.76 (0.72~0.8) | <0.001 |
| Smoking before pregnancy, n (%) | | |
| No | 1(Ref) |  |
| Yes | 1.27 (1.09~1.47) | 0.002 |
| Insurance, n (%) |  |  |
| Medicaid | 1(Ref) |  |
| Private Insurance | 0.72 (0.68~0.77) | <0.001 |
| Self-Pay | 0.62 (0.53~0.72) | <0.001 |
| Other | 0.86 (0.78~0.96) | 0.004 |
| Parity before the current pregnancy, n (%) | | |
| 0 | 1(Ref) |  |
| ≥ 1 | 0.86 (0.84~0.88) | <0.001 |
| Previous termination or fetal loss, n (%) | | |
| No | 1(Ref) |  |
| Yes | 1.14 (1.11~1.18) | <0.001 |
| Previous neonatal death, n (%) | | |
| No | 1(Ref) |  |
| Yes | 1.96 (1.76~2.19) | <0.001 |
| Previous preterm birth, n (%) |  |  |
| No | 1(Ref) |  |
| Yes | 3.05 (2.89~3.22) | <0.001 |
| Previous cesarean section, n (%) | | |
| No | 1(Ref) |  |
| Yes | 1.16 (1.12~1.21) | <0.001 |
| Pre-pregnancy diabetes, n (%) | | |
| No | 1(Ref) |  |
| Yes | 2.83 (2.59~3.09) | <0.001 |
| Pre-pregnancy hypertension, n (%) | | |
| No | 1(Ref) |  |
| Yes | 2.59 (2.46~2.73) | <0.001 |
| Pre-pregnancy BMI, n (%) |  |  |
| < 18.5 | 1.13 (1.01~1.26) | 0.034 |
| 18.5 - 24.9 | 1(Ref) |  |
| 25.0 - 29.9 | 1.33 (1.28~1.37) | <0.001 |
| 30.0 - 34.9 | 1.68 (1.62~1.75) | <0.001 |
| 35.0 - 39.9 | 1.94 (1.84~2.04) | <0.001 |
| ≥ 40.0 | 2.17 (2.04~2.31) | <0.001 |

**Abbreviations:** BMI, body mass index; PTB, preterm birth; OR, odds ratio; CI, confidence interval; Ref, reference.

**Supplemental table 4. Multinomial logistic regression of pre-pregnancy BMI and PTB.**

| Pre-pregnancy BMI | n. Total | n. Ref % | Preterm birth categories | | | | | | | | |
| --- | --- | --- | --- | --- | --- | --- | --- | --- | --- | --- | --- |
|  |  |  | Moderate to late preterm birth,  n. Event %, OR (95% CI), P-value | | | Very preterm birth,  n. Event %, OR (95% CI), P-value | | | Extremely preterm birth,  n. Event %, OR (95% CI), P-value | | |
| < 18.5 | 3643 | 3274 (89.9) | 325 (8.9) | 1.15 (1.02~1.29) | 0.021 | 27 (0.7) | 0.97 (0.66~ 1.42) | 0.861 | 17 (0.5) | 1.34 (0.82~ 2.18) | 0.248 |
| 18.5 - 24.9 | 89603 | 81454 (90.9) | 7175 (8.0) | 1(Ref) |  | 677 (0.8) | 1(Ref) |  | 297 (0.3) | 1(Ref) |  |
| 25.0 - 29.9 | 53248 | 47010 (88.3) | 5346 (10.0) | 1.23 (1.18~1.27) | <0.001 | 551 (1.0) | 1.32 (1.18~ 1.48) | <0.001 | 341 (0.6) | 1.81 (1.55~ 2.12) | <0.001 |
| 30.0 - 34.9 | 28686 | 24556 (85.6) | 3464 (12.1) | 1.44 (1.38~1.50) | <0.001 | 410 (1.4) | 1.76 (1.55~ 2.00) | <0.001 | 256 (0.9) | 2.40 (2.02~ 2.85) | <0.001 |
| 35.0 - 39.9 | 14195 | 11890 (83.8) | 1922 (13.5) | 1.56 (1.48~1.65) | <0.001 | 236 (1.7) | 1.96 (1.68~ 2.28) | <0.001 | 147 (1.0) | 2.66 (2.17~ 3.27) | <0.001 |
| ≥ 40.0 | 7862 | 6461 (82.2) | 1173 (14.9) | 1.68 (1.57~1.80) | <0.001 | 143 (1.8) | 2.08 (1.72~ 2.51) | <0.001 | 85 (1.10) | 2.73 (2.12~ 3.51) | <0.001 |

Adjusted for age of the pregnancy, race, marital status, education level, smoking before pregnancy, insurance, parity before the current pregnancy, previous termination or fetal loss, previous neonatal death, previous PTB, previous cesarean section, pre-pregnancy diabetes, and pre-pregnancy hypertension.

**Abbreviations:** BMI, body mass index; PTB, preterm birth; OR, odds ratio; CI, confidence interval; Ref, reference.

**Supplemental table 5. Subgroup analyses between pre-pregnancy BMI and PTB by previous termination or fetal loss.**

| Subgroup | n. Total | n. Event % | Crude OR (95%CI) | Crude P value | Adjusted OR (95%CI) | Adjusted P value | P for interaction |
| --- | --- | --- | --- | --- | --- | --- | --- |
| Previous termination or fetal loss | | | | | | | 0.002 |
| Yes | 76684 | 9434 (12.3) |  |  |  |  |  |
| < 18.5 | 1260 | 126 (10.0) | 1.04 (0.86~1.25) | 0.705 | 1.06 (0.88~1.28) | 0.549 |  |
| 18.5 - 24.9 | 33663 | 3258 (9.7) | 1(Ref) |  | 1(Ref) |  |  |
| 25.0 - 29.9 | 20916 | 2537 (12.1) | 1.29 (1.22~1.36) | <0.001 | 1.20 (1.14~1.27) | <0.001 |  |
| 30.0 - 34.9 | 11678 | 1773 (15.2) | 1.67 (1.57~1.78) | <0.001 | 1.47 (1.38~1.57) | <0.001 |  |
| 35.0 - 39.9 | 5858 | 1060 (18.1) | 2.06 (1.91~2.22) | <0.001 | 1.70 (1.57~1.84) | <0.001 |  |
| ≥ 40.0 | 3309 | 680 (20.6) | 2.41 (2.20~2.65) | <0.001 | 1.95 (1.77~2.14) | <0.001 |  |
| No | 120553 | 13158 (10.9) |  |  |  |  |  |
| < 18.5 | 2383 | 243 (10.2) | 1.19 (1.03~1.36) | 0.014 | 1.18 (1.03~1.36) | 0.015 |  |
| 18.5 - 24.9 | 55940 | 4891 (8.7) | 1(Ref) |  | 1(Ref) |  |  |
| 25.0 - 29.9 | 32332 | 3701 (11.4) | 1.35 (1.29~1.41) | <0.001 | 1.29 (1.23~1.35) | <0.001 |  |
| 30.0 - 34.9 | 17008 | 2357 (13.9) | 1.68 (1.59~1.77) | <0.001 | 1.52 (1.44~1.61) | <0.001 |  |
| 35.0 - 39.9 | 8337 | 1245 (14.9) | 1.83 (1.71~1.96) | <0.001 | 1.59 (1.48~1.71) | <0.001 |  |
| ≥ 40.0 | 4553 | 721 (15.8) | 1.96 (1.80~2.14) | <0.001 | 1.60 (1.47~1.75) | <0.001 |  |

Adjusted for age of the pregnancy, race, marital status, education level, smoking before pregnancy, insurance, parity before the current pregnancy, previous neonatal death, previous PTB, previous cesarean section, pre-pregnancy diabetes, and pre-pregnancy hypertension.

**Abbreviations:** BMI, body mass index; PTB, preterm birth; OR, odds ratio; CI, confidence interval; Ref, reference.

**Supplemental table 6. Sensitivity analysis for multinomial logistic regression of pre-pregnancy BMI and PTB.**

| Variable | n. Total | n. Ref % | Preterm birth categories | | | | | |
| --- | --- | --- | --- | --- | --- | --- | --- | --- |
|  |  |  | Moderate to late preterm birth,  OR (95% CI), P-value | | Very preterm birth,  OR (95% CI), P-value | | Extremely preterm birth,  OR (95% CI), P-value | |
| **Subjects without previous preterm birth (n=190222)** | | | | | | | | |
| < 18.5 | 3535 | 3194 (90.4) | 1.13 (1.00~1.28) | 0.046 | 1.07 (0.72~ 1.57) | 0.748 | 1.40 (0.86~ 2.29) | 0.181 |
| 18.5 - 24.9 | 86818 | 79308 (91.3) | 1(Ref) |  | 1(Ref) |  | 1(Ref) |  |
| 25.0 - 29.9 | 51285 | 45581 (88.9) | 1.23 (1.18~1.28) | <0.001 | 1.37 (1.22~ 1.55) | <0.001 | 1.80 (1.53~ 2.12) | <0.001 |
| 30.0 - 34.9 | 27456 | 23717 (86.4) | 1.45 (1.38~1.51) | <0.001 | 1.83 (1.60~ 2.08) | <0.001 | 2.34 (1.96~ 2.80) | <0.001 |
| 35.0 - 39.9 | 13611 | 11501 (84.5) | 1.58 (1.49~1.67) | <0.001 | 2.07 (1.76~ 2.43) | <0.001 | 2.61 (2.10~ 3.23) | <0.001 |
| ≥ 40.0 | 7517 | 6233 (82.9) | 1.69 (1.58~1.82) | <0.001 | 2.24 (1.84~ 2.72) | <0.001 | 2.73 (2.10~ 3.54) | <0.001 |
| **Subjects without pre-pregnancy diabetes (n=194580)** | | | | | | | | |
| < 18.5 | 3628 | 3260 (89.9) | 1.16 (1.03~1.30) | 0.016 | 0.98 (0.67~ 1.45) | 0.924 | 1.35 (0.83~ 2.21) | 0.229 |
| 18.5 - 24.9 | 89121 | 81076 (91) | 1(Ref) |  | 1(Ref) |  | 1(Ref) |  |
| 25.0 - 29.9 | 52615 | 46534 (88.4) | 1.22 (1.18~1.27) | <0.001 | 1.35 (1.20~ 1.51) | <0.001 | 1.81 (1.54~ 2.12) | <0.001 |
| 30.0 - 34.9 | 28056 | 24093 (85.9) | 1.44 (1.38~1.51) | <0.001 | 1.78 (1.57~ 2.03) | <0.001 | 2.41 (2.03~ 2.87) | <0.001 |
| 35.0 - 39.9 | 13672 | 11529 (84.3) | 1.57 (1.48~1.66) | <0.001 | 1.95 (1.66~ 2.29) | <0.001 | 2.71 (2.20~ 3.33) | <0.001 |
| ≥ 40.0 | 7488 | 6197 (82.8) | 1.70(1.58~1.83) | <0.001 | 2.19 (1.81~ 2.66) | <0.001 | 2.67 (2.06~ 3.46) | <0.001 |
| **Subjects without pre-pregnancy hypertension (n=188533)** | | | | | | | | |
| < 18.5 | 3616 | 3251 (89.9) | 1.16 (1.03~1.31) | 0.012 | 1.00 (0.68~ 1.47) | 0.982 | 1.39 (0.85~ 2.28) | 0.186 |
| 18.5 - 24.9 | 88190 | 80361 (91.1) | 1(Ref) |  | 1(Ref) |  | 1(Ref) |  |
| 25.0 - 29.9 | 51172 | 45406 (88.7) | 1.23 (1.19~1.28) | <0.001 | 1.32 (1.18~ 1.49) | <0.001 | 1.84 (1.56~ 2.17) | <0.001 |
| 30.0 - 34.9 | 26551 | 22973 (86.5) | 1.45 (1.38~1.52) | <0.001 | 1.79 (1.57~ 2.04) | <0.001 | 2.49 (2.08~ 2.99) | <0.001 |
| 35.0 - 39.9 | 12501 | 10621 (85) | 1.61 (1.52~1.71) | <0.001 | 2.08 (1.76~ 2.45) | <0.001 | 2.80 (2.25~ 3.50) | <0.001 |
| ≥ 40.0 | 6503 | 5420 (83.3) | 1.83 (1.69~1.97) | <0.001 | 2.18 (1.77~ 2.70) | <0.001 | 3.08 (2.34~ 4.05) | <0.001 |
| **Subjects without previous preterm birth, pre-pregnancy diabetes, pre-pregnancy hypertension (n=180154)** | | | | | | | | |
| < 18.5 | 3497 | 3159 (90.3) | 1.16 (1.02~1.31) | 0.021 | 1.12 (0.76~ 1.65) | 0.572 | 1.47 (0.90~ 2.41) | 0.124 |
| 18.5 - 24.9 | 85053 | 77912 (91.6) | 1(Ref) |  | 1(Ref) |  | 1(Ref) |  |
| 25.0 - 29.9 | 48839 | 43661 (89.4) | 1.23 (1.18~1.28) | <0.001 | 1.42 (1.25~ 1.60) | <0.001 | 1.82 (1.54~ 2.16) | <0.001 |
| 30.0 - 34.9 | 25045 | 21888 (87.4) | 1.46 (1.39~1.53) | <0.001 | 1.89 (1.64~ 2.17) | <0.001 | 2.43 (2.01~ 2.93) | <0.001 |
| 35.0 - 39.9 | 11691 | 10046 (85.9) | 1.63 (1.53~1.73) | <0.001 | 2.18 (1.83~ 2.60) | <0.001 | 2.87 (2.28~ 3.61) | <0.001 |
| ≥ 40.0 | 6029 | 5086 (84.4) | 1.86 (1.72~2.01) | <0.001 | 2.45 (1.96~ 3.06) | <0.001 | 3.04 (2.28~ 4.07) | <0.001 |
| **Subjects with a pre-pregnancy BMI of mean ± 3SD (n=195180)** | | | | | | | | |
| < 18.5 | 3643 | 3274 (89.9) | 1.15 (1.02~1.29) | 0.021 | 0.97 (0.66~ 1.42) | 0.864 | 1.33 (0.82~ 2.18) | 0.249 |
| 18.5 - 24.9 | 89603 | 81454 (90.9) | 1(Ref) |  | 1(Ref) |  | 1(Ref) |  |
| 25.0 - 29.9 | 53248 | 47010 (88.3) | 1.23 (1.18~1.27) | <0.001 | 1.32 (1.18~ 1.48) | <0.001 | 1.81 (1.55~ 2.12) | <0.001 |
| 30.0 - 34.9 | 28686 | 24556 (85.6) | 1.44 (1.37~1.50) | <0.001 | 1.76 (1.55~ 2.00) | <0.001 | 2.38 (2.01~ 2.83) | <0.001 |
| 35.0 - 39.9 | 14195 | 11890 (83.8) | 1.56 (1.47~1.65) | <0.001 | 1.95 (1.67~ 2.28) | <0.001 | 2.64 (2.15~ 3.24) | <0.001 |
| ≥ 40.0 | 5805 | 4791 (82.5) | 1.64 (1.52~1.78) | <0.001 | 1.96 (1.57~ 2.44) | <0.001 | 3.17 (2.43~ 4.14) | <0.001 |

Age of the pregnancy, race, marital status, education level, smoking before pregnancy, insurance, parity before the current pregnancy, previous termination or fetal loss, previous neonatal death, previous preterm birth, previous cesarean section, pre-pregnancy diabetes, and pre-pregnancy hypertension were adjusted for in the model except when the variable was excluded.

**Abbreviations:** BMI, body mass index; PTB, preterm birth; OR, odds ratio; CI, confidence interval; Ref, reference.
